# Explainable AI–Driven Comparative Analysis of Machine Learning Models for Predicting HIV Viral Nonsuppression in Ugandan Patients: Retrospective Cross-Sectional Study

**DOI:** 10.2196/68196

**Published:** 2026-01-06

**Authors:** Francis Ngema, Albert Whata, Micheal O Olusanya, Siyabonga Mhlongo

**Affiliations:** 1 Centre of Applied Data Science University of Johannesburg Johannesburg South Africa; 2 Department of Statistics University of Pretoria Pretoria South Africa; 3 Department of Computer Science and Information Technology Sol Plaatje University Kimberley South Africa; 4 Department of Applied Information Systems University of Johannesburg Johannesburg South Africa

**Keywords:** HIV viral suppression, machine learning, explainable AI, artificial intelligence, antiretroviral therapy, adherence, Uganda, predictive modeling, XGBoost, extreme gradient boosting, clinical decision-making, public health

## Abstract

**Background:**

HIV viral suppression is essential for improving health outcomes and reducing transmission rates among people living with HIV. In Uganda, where HIV/AIDS is a major public health concern, machine learning (ML) models can predict viral suppression effectively. However, the limited use of explainable artificial intelligence (XAI) methods affects model transparency and clinical utility.

**Objective:**

This study aimed to develop and compare ML models for predicting viral nonsuppression in Ugandan people living with HIV on antiretroviral therapy (ART), and then systematically apply comprehensive XAI techniques to the best-performing model to identify key predictors and demonstrate interpretability at both population and individual patient levels.

**Methods:**

We retrospectively analyzed clinical and demographic data from 1101 Ugandan people living with HIV on ART at the HIV clinic in Muyembe Health Centre IV between June 2016 and April 2018, focusing on predicting viral nonsuppression (viral load >1000 copies per milliliter). The dataset was divided into model-building (training: 80%) and validation (test: 20%) sets. To address class imbalance, the synthetic minority over-sampling technique was applied. For global explanation, 8 ML algorithms—logistic regression, stacked ensemble, random forest, support vector machines, extreme gradient boosting (XGBoost), k-nearest neighbors, naïve Bayes, and artificial neural networks—were compared. Model performance was evaluated using metrics such as accuracy, precision, recall, *F*_1_-score, Cohen κ, and area under the curve (AUC). For local explanation, individual conditional expectation plots, Shapley Additive Explanations (SHAP), breakdown, and SHAP force plots were used to provide insights into predictions for individual patients.

**Results:**

The XGBoost ensemble model demonstrated superior performance with an accuracy of 0.89, precision of 0.59, recall of 0.65, and AUC of 0.80. The model achieved high specificity (0.93) and moderate sensitivity, yielding a Cohen κ of 0.55 and *F*_1_-score of 0.62, indicating good discriminative ability for viral nonsuppression prediction. SHAP feature importance analysis identified adherence assessment over the preceding 3 months as the most influential predictor of viral nonsuppression, followed by age group, urban residence, and duration on ART. Local SHAP consistently demonstrated that poor adherence was the primary driver of both correctly identified nonsuppressed cases and false positive predictions, reinforcing adherence as the critical determinant of treatment outcomes.

**Conclusions:**

The XGBoost model demonstrated optimal performance for predicting viral nonsuppression among Ugandan people living with HIV on ART, achieving an AUC of 0.80. Comprehensive XAI analysis identified adherence assessment as the primary predictor, followed by age group, residence type, and ART duration. XAI methods provided transparent interpretation of model predictions at both population and individual patient levels, enabling identification of key risk factors for targeted clinical interventions in resource-limited settings.

## Introduction

HIV/AIDS remains a major public health issue in Uganda, with an estimated 1.4 million people living with the virus and an adult prevalence of 5.2%. According to the most recent estimates, approximately 93% of individuals living with HIV in Uganda are currently receiving antiretroviral therapy (ART) [1,2]. Despite challenges, progress is evident with 1.2 million individuals on antiretroviral treatment and a 44% reduction in new infections since 2010. Significant strides have been made in reducing pediatric HIV infections by 61%, though vertical transmission rates after breastfeeding remain at 8.6%. Continued efforts are essential to meet the goal of ending AIDS as a public health threat by 2030 [1-3].

Viral load monitoring remains a crucial component of ART success due to its early detection of treatment failure, enabling timely interventions to address adherence issues or drug resistance [1]. It distinguishes between true drug resistance and temporary adherence lapses, allowing for targeted interventions without unnecessary medication changes [4]. In addition, public health officials can evaluate program effectiveness and identify areas for improvement by tracking trends in viral suppression rates. Achieving viral suppression, defined by the World Health Organization (WHO) as an HIV viral load <1000 copies per milliliter, is the primary goal of ART for people living with HIV. This public health threshold, used for global monitoring and in resource-limited settings, differs from clinical thresholds used in high-income countries (<200 or <50 copies per milliliter for “undetectable” status) [5-8]. This crucial milestone not only significantly reduces the risk of transmitting HIV to sexual partners but also minimizes the risk of mother-to-child transmission during breastfeeding. However, predicting and achieving viral suppression can be challenging due to the complex interplay of factors beyond adherence to ART medication. Research suggests that factors such as age, sex, sociodemographic characteristics, clinical, treatment, and potentially psychological factors also play a role in influencing treatment success [9-11]. Consequently, there is growing interest in using machine learning (ML) models to enhance prediction accuracy.

ML analyzes complex, high-dimensional data and captures complex relationships between variables [12]. Rajula et al [12] further state that this capability is valuable in HIV viral suppression prediction, where traditional statistical methods often struggle with this type of data, potentially overlooking crucial factors influencing viral failure risk. Several studies in Eastern and Southern Africa have demonstrated the potential of ML algorithms such as random forest and logistic regression for predicting viral suppression in HIV [13-18]. For instance, Mamo et al [18] demonstrated the potential of ML approaches, achieving an area under the curve (AUC) of 0.9989 for viral failure prediction using random forest with a comprehensive methodology including cross-validation and imbalanced data handling. While these results are promising, the near-perfect performance highlights the need for external validation studies to establish realistic performance benchmarks and confirm the generalizability of ML models in diverse HIV care settings.

Despite significant advancements in ML for predicting HIV viral suppression, the adoption of explainable artificial intelligence (XAI) techniques, which provide transparent insights into how models make predictions, remains limited within this domain [19]. Our study hypothesized that comprehensive XAI techniques could be successfully integrated with ML models to provide interpretable predictions for HIV viral suppression in a resource-limited setting, identifying key risk factors at both population and individual patient levels. This gap presents a critical opportunity for improvement through the implementation of local and global interpretability methods.

Our study addressed this limitation by developing and comparing ML models for HIV viral suppression prediction in Ugandan people living with HIV, and then systematically applying comprehensive XAI techniques to enhance model interpretability. Multiple ML algorithms were built and compared, XAI methods were applied to the best-performing model to identify key predictive factors, and interpretability was demonstrated at both population and individual patient levels. This integrated approach combined predictive accuracy with transparent model interpretation, providing actionable insights for clinical decision-making in resource-limited settings.

## Methods

This section outlines the methodological approach used to achieve our research objectives (Multimedia Appendix 1).

### Study Design

This study conducted a secondary analysis of a retrospective cohort dataset originally collected by Wakooko et al [11], who used traditional binary logistic regression analysis. The original study reviewed clinical records of people living with HIV on ART for at least 6 months at Muyembe Health Centre IV (HCIV), the primary ART site in Bulambuli District, Uganda. In contrast to the original analysis, this study used ML approaches to develop predictive models for viral suppression outcomes. Furthermore, XAI techniques were applied to the best-performing model to provide insights into the factors influencing viral suppression, enhancing both model interpretability and transparency in the clinical decision-making process.

### Study Setting

This study used a dataset collected in Bulambuli District, located in Eastern Uganda, with Muyembe HCIV serving as the sole data source. Although the district comprises 10 Health Centre IIIs and 1 HCIV, Muyembe HCIV functions as the district’s primary and fully operational ART site. It maintains the most complete ART records and provides centralized HIV care for the area (Multimedia Appendix 2).

### Study Population, Sampling, and Data Acquisition

**Figure 1:**
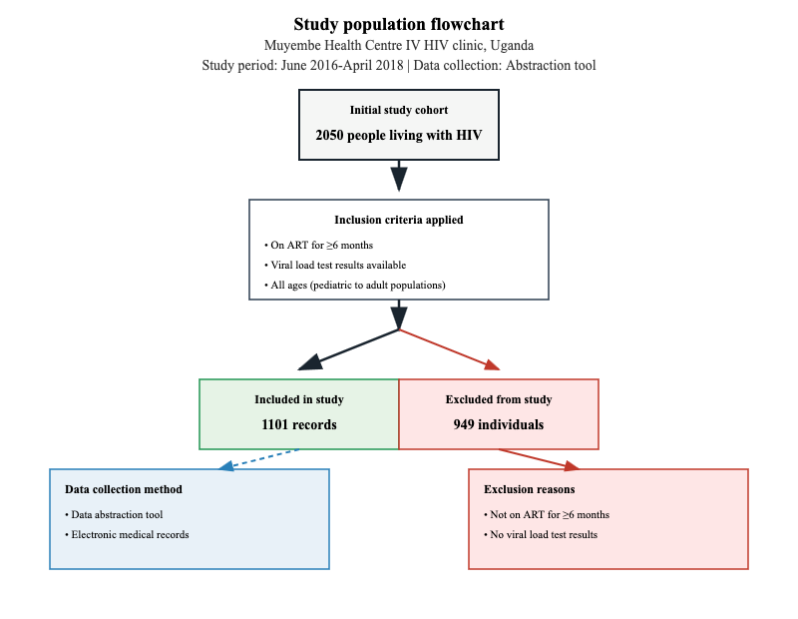
Flowchart of participant selection for the study of people living with HIV on ART at Muyembe Health Centre IV, June 2016 to April 2018. ART: antiretroviral therapy.

This study used a secondary dataset sourced from the Mendeley data repository [20], comprising information extracted from medical records of people living with HIV who received ART at Muyembe HCIV between June 2016 and April 2018. The study population consisted of people living with HIV enrolled in care at Muyembe HCIV during the study period. From an initial cohort of 2050 people living with HIV enrolled at the facility, 1101 participants met the inclusion criteria and were included in the final study sample. A total of 949 individuals were excluded for not meeting the inclusion criteria (Figure 1).

The dataset included demographic, clinical, and treatment-related variables of people living with HIV receiving ART. Variables such as age, sex, WHO clinical stage at ART initiation, ART regimen, cluster of differentiation 4 (CD4) count at ART initiation, adherence assessment, and treatment duration were incorporated into the analysis (Table S2 in Multimedia Appendix 3 provides comprehensive mapping between variable descriptions, code names, and original data codes). Adherence assessment was conducted by reviewing patient treatment cards, which contained documented records of medication adherence over the preceding 3 months as recorded by health care providers during routine clinic visits.

The primary outcome of interest in this study was viral nonsuppression, defined as a viral load greater than 1000 copies per milliliter, consistent with Uganda’s national antiretroviral treatment monitoring guidelines [6]. To facilitate model predictions and subsequent clinical interpretation, viral load measurements were dichotomized, whereby viral nonsuppression was assigned a value of 1 (positive class), and viral suppression was assigned a value of 0.

### Inclusion and Exclusion Criteria

Participants were eligible for inclusion if they were on ART for 6 months or longer and had viral load test results available. All ages were included, covering a wide range of people living with HIV from pediatric to adult populations.

### Data Preparation and Preprocessing

The dataset was randomly partitioned into training (80%) and testing (20%) subsets, with stratification to preserve the outcome variable distribution (viral suppression status). We assessed missingness patterns across all 27 variables initially extracted from the dataset (Table S1 in Multimedia Appendix 3). Factor levels were harmonized across subsets, and variables with more than 80% missingness (specific other medication, reason for stopping ART, and specific opportunistic infection) were excluded. All preprocessing steps, including imputation, were performed exclusively on the training dataset to prevent data leakage and ensure unbiased model evaluation. Remaining features underwent systematic cleaning, including mean imputation for numeric variables and mode imputation for categorical predictors, with clinically informed handling of missing values through “unknown” categories for marital status and supporter relationships to preserve potential clinical significance of missingness patterns. Ordinal variables (eg, WHO clinical stage, age group, ART duration, adherence assessment, weight, and time before viral load testing) were encoded as ordered factors, ensuring that clinically meaningful ordering was preserved. Nominal categorical variables (eg, sex, marital status, residence type, opportunistic infection history, tuberculosis history on ART, point of entry in ART clinic, ART history, ART supporter presence, supporter relationship, reported side effects, dosing frequency, and pre-ART counseling status) were harmonized across datasets, aligned to consistent reference categories, and subsequently one-hot encoded using dummy variables. The derived categorical variable CD4 lymphocyte count category was removed in favor of retaining the original continuous CD4 lymphocyte count at ART initiation.

Two distinct preprocessing pipelines were implemented. The first did not apply any class-imbalance technique and relied solely on structured preprocessing steps using the recipes package (dummy encoding, normalization, ordinal scoring, and zero-variance removal). The second pipeline addressed class imbalance by applying the synthetic minority over-sampling technique (SMOTE) to the training data, followed by support vector machine-recursive feature elimination (SVM-RFE) for feature selection of predictors. Feature set sizes varied across model implementations: the final dataset contained 20 features, preprocessing expanded this to 25 features for selected models, while other feature selection approaches yielded reduced sets of 13 features for the extreme gradient boosting (XGBoost) model. All augmentation and feature selection procedures were applied exclusively to training data, preserving test set integrity and enabling systematic evaluation of different preprocessing strategies while maintaining fully standardized, reproducible, and leakage-free datasets suitable for downstream model development.

### Model Training and Tuning

This section outlines the key steps undertaken to develop ML models for predicting HIV viral suppression among patients receiving ART in Uganda. The following subsections describe model building and model performance and evaluation.

#### Model Building

A diverse set of ML models was developed to predict viral nonsuppression status, including random forest, XGBoost, artificial neural networks, support vector machines, logistic regression, k-nearest neighbors, naïve Bayes, and a stacked ensemble with random forest and XGBoost base learners and an XGBoost meta-learner. Stacked ensembles are a 2-level modeling strategy that harnesses the strengths of multiple ML models [21,22]. Model development used nested 10-fold cross-validation to optimize hyperparameters and minimize overfitting. Each algorithm was trained using structured hyperparameter grids. These models were chosen based on their proven effectiveness in classification tasks and their ability to handle complex relationships within the data.

#### Model Performance and Evaluation

Evaluation incorporated a comprehensive set of metrics, including accuracy, precision, recall (sensitivity), specificity, *F*_1_-score, Cohen κ, and AUC. Performance was assessed through both internal cross-validation and independent test sets, enabling robust benchmarking of the models. In addition, feature importance and the stability of selected predictors were examined to compare model behavior across the imbalanced and SMOTE-SVM-RFE pipelines.

Recall was prioritized due to its clinical importance in identifying patients at high risk for failing to achieve viral suppression. The *F*_1_-score offered a balanced view of precision and recall, particularly useful for imbalanced datasets. Cohen κ accounted for the possibility of agreement occurring by chance, providing a more robust measure than simple accuracy. The receiver operating characteristic curve visually represented the trade-off between true positive (TP) rates and false positive (FP) rates, with the AUC quantifying the model’s discriminative ability.

Following model training, the optimal classification threshold was determined using the Youden *J* statistic (sensitivity+specificity–1) on the training set receiver operating characteristic curve [23]. This approach maximizes the combined sensitivity and specificity and represents a posttraining internal validation step that does not influence model fitting. The resulting threshold was held fixed and applied unchanged to the independent test set for all performance metric calculations and confusion matrix computation.

Probability calibration was performed using isotonic regression fitted on the training set predictions [24]. The fitted calibration function was then applied to the independent test set to generate calibrated probability estimates. A calibration plot was created for the best-performing model to evaluate alignment between predicted probabilities and actual outcomes. Brier scores were calculated to quantify the accuracy of probabilistic predictions before and after calibration [25].

### Interpretation Methods

Our research used a multifaceted approach to interpret the best-performing model used for HIV viral suppression prediction**.** This approach combined global and local interpretability techniques to understand how features influenced the model’s decisions.

#### Global Explanation

We incorporated global Shapley Additive Explanations (SHAP) for interpretability, which assigned attribution values to each feature, explaining its contribution to specific predictions [26]. SHAP summary plots helped identify the most important global features influencing the best model’s predictions. Furthermore, we used dependence plots to visualize the average effect of individual features on predictions, showing how the predicted outcome (eg, viral nonsuppression) changed in response to variations in each feature [27].

#### Local Explanation

Local interpretability techniques examined the reasoning behind individual predictions [19]. Individual conditional expectations (ICEs) were used to illustrate how changes in a single feature, while holding others constant, impacted predictions for each participant [28,29]. Breakdown plots further decomposed predictions into contributions from individual features, visualizing their influence on specific predictions [29,30]. In addition, SHAP was used to explore feature interactions and their influence on individual predictions, providing deeper insight into local model reasoning.

### Software and Analytical Tools

The analysis was conducted on a machine with the following specifications: graphics: Intel Iris Plus Graphics 1536 MB, RAM: 16 GB 3733 MHz LPDDR4X, and processor: 2 GHz Quad-Core Intel Core i5, running macOS Sonoma (version 14.6.1; 23G93). The programming languages used include Python (version 3.9; Python Software Foundation) and R (version 4.3.3, 202-02-29, “Angel Food Cake”; R Foundation for Statistical Computing), with RStudio 2024.09.0+375 (Posit Software, PBC) serving as the integrated development environment for both R and Python, while Stata 18 SE (StataCorp LLC) was used to import and perform preliminary descriptive analyses on the raw dataset, which was provided in Stata’s proprietary .dta file format. The *RStata* package was used to import and describe the data in R.

Python integration was achieved via the *reticulate* package, using pandas for data manipulation. In R, *dplyr* was used for cleaning and renaming columns, improving data clarity. Data wrangling and preprocessing were conducted using a suite of R packages. The *dplyr* package was used for data manipulation tasks, such as filtering, mutating, and summarizing data. The tidymodels framework was used for recipe creation and model baking. ML models were trained and evaluated using the *caret* package, supporting hyperparameter tuning and cross-validation. To ensure interpretability, a suite of XAI packages—*iml*, *vip*, *pdp*, *breakDown*, *SHAPforxgboost*, and *DALEX*—was used, providing tools for variable importance, partial dependence plots, breakdown plots, and SHAP [19].

### Ethical Considerations

The original study, titled “Viral Load Suppression and Associated Factors among HIV Patients on Antiretroviral Treatment in Bulambuli District, Eastern Uganda: A Retrospective Cohort Study” by Wakooko et al [11], received ethics approval from both the Busitema University Faculty of Health Sciences Higher Degrees and Research Committee and the Mbale Regional Referral Hospital Research and Ethics Committee (Ref: MRRH-REC-IN-COM 081/2018). Permission to conduct the study was further obtained from the Bulambuli District Health Office. A waiver of informed consent was granted, as the study involved secondary analysis of existing medical records initially collected for routine patient care. Participant privacy and confidentiality were maintained through deidentification procedures: the data abstraction tool used numerical identifiers rather than names, ensuring that no individual personal data were exposed, and all collected data were stored securely with access restricted to research personnel. No compensation was provided to participants, as no direct participant contact occurred. The research presented no risk of harm to participants. For this current secondary analysis study, ethics approval was granted by the School of Consumer Intelligence and Information Systems Research Ethics Committee of the University of Johannesburg (approval: 2024SCiiS029).

## Results

This section presents the findings from our analysis of the ML models developed to predict HIV viral suppression among Ugandan people living with HIV receiving ART.

### Clinical and Demographic Profile

This study analyzed baseline sociodemographic, clinical, and biomarker data to understand factors influencing viral suppression among patients receiving ART in Uganda. A detailed breakdown of these features stratified by viral suppression status is presented (Table 1).

**Table 1:** Baseline sociodemographics, clinical factors, and biomarkers of people living with HIV on antiretroviral therapy (ART) in a retrospective cohort study in 2019, Bulambuli District, Uganda.

Predictors			Total (N=1101)		Suppressed <1000 RNA copies per milliliter (n=944)		Not suppressed >1000 RNA copies per milliliter (n=157)
**Age group (years)**							
	0-5	24 (2.2)		20 (2.1)		4 (2.5)	
	6-12	69 (6.3)		50 (5.3)		19 (12.1)	
	13-19	41 (3.7)		28 (3)		13 (8.3)	
	20-35	434 (39.4)		372 (39.4)		62 (39.5)	
	Above 35	533 (48.4)		474 (50.2)		59 (37.6)	
**Sex**							
	Male	334 (30.3)		289 (30.6)		45 (28.7)	
	Female	767 (69.7)		655 (69.4)		112 (71.3)	
**Marital status**							
	Single	301 (27.3)		237 (25.1)		64 (40.8)	
	Married	713 (64.8)		629 (66.6)		84 (53.5)	
	Divorced	87 (7.9)		78 (8.3)		9 (5.7)	
**Residence type**							
	Rural	678 (61.6)		588 (62.3)		90 (57.3)	
	Urban	423 (38.4)		356 (37.7)		67 (42.7)	
**Adherence assessment last 3 months**							
	Poor <80%	78 (7.1)		19 (2)		59 (37.6)	
	Fair 80%-95%	147 (13.4)		96 (10.2)		51 (32.5)	
	Good >95%	876 (79.6)		829 (87.8)		47 (29.9)	
**WHO^a^ clinical stage at ART initiation**							
	Stage 1	239 (21.7)		200 (21.2)		39 (24.8)	
	Stage 2	540 (49)		462 (48.9)		78 (49.7)	
	Stage 3	313 (28.4)		273 (28.9)		40 (25.5)	
	Stage 4	9 (0.8)		9 (1)		0 (0)	
**Weight at ART initiation (kg)**							
	1-20	70 (6.4)		56 (5.9)		14 (8.9)	
	21-50	489 (44.4)		413 (43.8)		76 (48.4)	
	Above 50	542 (49.2)		475 (50.3)		67 (42.7)	
**Opportunistic infection history**							
	Yes	80 (7.3)		68 (7.2)		12 (7.6)	
	No	1021 (92.7)		876 (92.8)		145 (92.4)	
**Tuberculosis history on ART**							
	Yes	15 (1.4)		12 (1.3)		3 (1.9)	
	No	1086 (98.6)		932 (98.7)		154 (98.1)	
**Point of entry in ART clinic**							
	OPD^b^	921 (83.7)		791 (83.8)		130 (82.8)	
	Maternity	139 (12.6)		120 (12.7)		19 (12.1)	
	Antenatal care service	41 (3.7)		33 (3.5)		8 (5.1)	
**Duration on ART (months)**							
	3-6	10 (0.9)		8 (0.8)		2 (1.3)	
	7-11	33 (3)		29 (3.1)		4 (2.5)	
	12-24	346 (31.4)		288 (30.5)		58 (36.9)	
	More than 24	712 (64.7)		619 (65.6)		93 (59.2)	
**ART history**							
	Yes	21 (1.9)		17 (1.8)		4 (2.5)	
	No	1080 (98.1)		927 (98.2)		153 (97.5)	
**Reported ART side effects**							
	Yes	88 (8)		71 (7.5)		17 (10.8)	
	No	1013 (92)		873 (92.5)		140 (89.2)	
**Frequency of ARV^c^ dosing**							
	Once	693 (62.9)		603 (63.9)		90 (57.3)	
	Twice	408 (37.1)		341 (36.1)		67 (42.7)	
**Pre-ART counseling status**							
	Yes	940 (85.4)		805 (85.3)		135 (86)	
	No	161 (14.6)		139 (14.7)		22 (14)	
**Treatment supporter presence**							
	Yes	1028 (93.4)		883 (93.5)		145 (92.4)	
	No	73 (6.6)		61 (6.5)		12 (7.6)	
**Treatment supporter relationship**							
	Care giver	224 (20.3)		196 (20.8)		28 (17.8)	
	Relative	606 (55)		516 (54.7)		90 (57.3)	
	Peer	13 (1.2)		11 (1.2)		2 (1.3)	
	Biological parent	87 (7.9)		66 (7)		21 (13.4)	
	Marriage partner	171 (15.5)		155 (16.4)		16 (10.2)	
**Time before viral load test on ART (months)**							
	6	110 (10%)		98 (10.4)		12 (7.6)	
	12	576 (52.3)		489 (51.8)		87 (55.4)	
	>12	415 (37.7)		357 (37.8)		58 (36.9)	
**Current ART regimen simplified**							
	TDF^d^-based	737 (66.9)		642 (68)		95 (60.5)	
	AZT^e^-based	325 (29.5)		271 (28.7)		54 (34.4)	
	ABC^f^-based	33 (3)		26 (2.8)		7 (4.5)	
	Other ART	6 (0.5)		5 (0.5)		1 (0.6)	
**CD4^g^ count category**							
	<200	801 (72.8)		687 (72.8)		114 (72.6)	
	200-500	225 (20.4)		194 (20.6)		31 (19.7)	
	>500	75 (6.8)		63 (6.7)		12 (7.6)	

^a^WHO: World Health Organization.

^b^OPD: outpatient department.

^c^ARV: antiretroviral.

^d^TDF: tenofovir disoproxil fumarate.

^e^AZT: zidovudine.

^f^ABC: abacavir.

^g^CD4: cluster of differentiation 4.

Among the 1101 people living with HIV on ART, 944 (85.7%) achieved viral suppression (<1000 RNA copies per milliliter). Four key demographic and clinical factors demonstrated notable patterns in relation to viral suppression outcomes.

Adherence patterns showed a strong association with viral suppression. Among participants with good adherence (>95%), 94.6% (829/876) achieved viral suppression compared to only 24.4% (19/78) of those with poor adherence (<80%). Fair adherence (80%-95%) resulted in 65.3% (96/147) suppression rates, demonstrating a clear adherence-response gradient. Age distribution revealed differential suppression rates across groups. Participants aged 35 years and older had the highest suppression rate at 89.9% (474/533), while adolescents (aged 13-19 years) showed the lowest at 68.3% (28/41). Children aged 6-12 years had a suppression rate of 72.5% (50/69), indicating age-related challenges in achieving optimal outcomes.

Duration on ART showed that established patients performed better, with 87% (619/712) of those on treatment >24 months achieving suppression compared to 83.2% (288/346) of patients treated for 12-24 months. Newer patients (3-11 months) had suppression rates of 86% (588/678). Residence type demonstrated urban-rural disparities, with rural residents achieving 86.7% (356/423) suppression compared to 84.2% (356/423) among urban residents, though this difference was modest.

### Global Explanation

The analysis included the performance evaluation of supervised learning classifiers, the assessment of feature importance, and the generation of dependence plots to illustrate the relationships between key features and model predictions.

#### Performance of Supervised Learning Classifiers

The imbalanced pipeline (Table 2) revealed substantial performance variation across algorithms, with neural networks achieving the highest accuracy (0.90) and precision (0.70), while k-nearest neighbors exhibited optimal recall (0.68). However, class imbalance severely impacted several algorithms, notably logistic regression, which achieved high precision (0.93) but critically low recall (0.13), rendering it clinically unsuitable for viral nonsuppression detection.

**Table 2:** Comparative performance of machine learning models on imbalanced data (train set: 882, test set: 219).

Model	Accuracy	Precision	Recall	Specificity	*F*_1_-score	κ	AUC^a^
XGBoost^b^	0.89	0.59	0.61	0.93	0.60	0.54	0.83
Stacked ensemble (XGBoost meta-learner)	0.88	0.58	0.61	0.93	0.59	0.52	0.77
Random forest	0.87	0.53	0.55	0.92	0.54	0.46	0.83
K-nearest neighbors	0.85	047	0.68	0.87	0.55	0.46	0.80
Logistic regression	0.69	0.93	0.13	0.79	0.1	—^c^	0.56
Naïve Bayes	0.86	—	—	1.00	—	—	0.75
SVM^d^	0.86	0.51	0.55	0.91	0.53	0.45	0.82
ANN^e^	0.90	0.70	0.51	0.96	0.59	0.53	0.78

^a^AUC: area under the curve.

^b^XGBoost: extreme gradient boosting.

^c^Not available.

^d^SVM: support vector machine.

^e^ANN: artificial neural network.

The SMOTE-balanced pipeline (Table 3) demonstrated improved recall across most algorithms, confirming the effectiveness of synthetic oversampling for addressing class imbalance. XGBoost achieved optimal overall performance with balanced metrics: accuracy (0.89), precision (0.59), recall (0.65), and robust agreement (κ=0.55).

**Table 3:** Comparative performance of machine learning models on SMOTEa-balanced and SVM-RFEb selected data (train set: 1008, test set: 219).

Model	Accuracy	Precision	Recall	Specificity	*F*_1_-score	κ	AUC^c^
XGBoost^d^	0.89	059	0.65	0.93	0.62	0.55	0.80
Stacked ensemble (XGBoost meta-learner)	0.74	0.31	0.71	0.74	0.44	0.30	0.76
Random forest	0.88	0.56	0.58	0.94	0.57	0.50	0.78
K-nearest neighbors	0.74	0.30	0.58	0.77	0.39	0.25	070
Logistic regression	0.79	0.36	0.68	0.80	0.47	0.35	0.82
Naïve Bayes	0.85	0.46	0.42	0.93	0.44	0.35	0.70
SVM^e^	0.87	0.60	0.29	0.97	0.39	0.33	0.67
ANN^f^	0.80	0.34	0.45	0.86	0.39	0.27	0.74

^a^SMOTE: synthetic minority over-sampling technique.

^b^SVM-RFE: support vector machine-recursive feature elimination.

^c^AUC: area under the curve.

^d^XGBoost: extreme gradient boosting.

^e^SVM: support vector machine.

^f^ANN: artificial neural network.

XGBoost emerged as the superior performer across both pipelines, demonstrating consistent excellence in ensemble learning principles. On the SMOTE-balanced dataset, XGBoost achieved clinically relevant performance with 65% sensitivity for viral nonsuppression detection while maintaining 93% specificity for correctly identifying suppressed patients. The model’s balanced *F*_1_-score (0.62) and substantial agreement (κ=0.55) indicate robust predictive capability suitable for clinical implementation. Feature selection via SVM-RFE enhanced model interpretability while preserving discriminative performance, yielding an AUC of 0.80 that meets clinically acceptable thresholds for viral suppression prediction. Cross-validation identified optimal hyperparameters at iteration 35. The model used the following hyperparameters: nrounds=35, max_depth=7, eta=0.1, gamma=1, colsample_bytree=0.8, min_child_weight=4, subsample=0.8, lambda=2.0, and scale_pos_weight=1.30 to address class imbalance. Threshold optimization yielded 0.611 for test evaluation to balance specificity and recall.

Isotonic regression calibration, fitted on training data and applied to the test set, substantially improved probability estimates. Calibration performance demonstrated marked improvement, with the Brier score decreasing from 0.1324 (uncalibrated) to 0.0739 (calibrated), representing a 44.2% reduction and indicating enhanced reliability of probability estimates. The calibrated model demonstrated enhanced discriminative performance, with AUC increasing modestly from 0.799 to 0.838 (Multimedia Appendix 4).

#### Feature Importance

**Figure 2:**
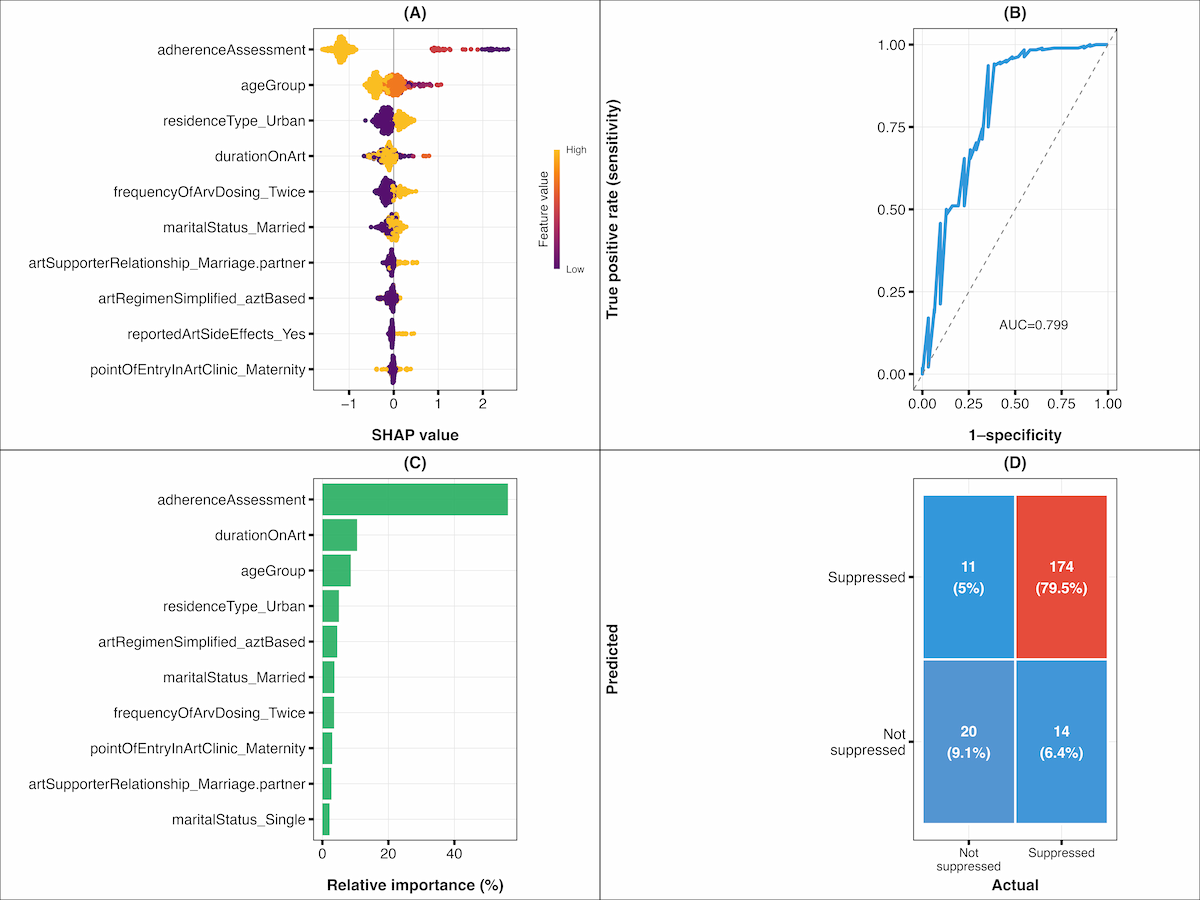
Model evaluation metrics for XGBoost classifier. (A) SHAP feature impact (beeswarm) illustrating feature influence on predictions, (B) AUC, (C) feature importance based on relative contribution, and (D) confusion matrix. AUC: area under the curve; SHAP: Shapley Additive Explanations; XGBoost: extreme gradient boosting.

SHAP analysis (Figure 2A) revealed differential feature impacts on viral nonsuppression predictions, with adherence assessment demonstrating the strongest influence on model decisions, followed by age group, residence type (urban), and duration on ART. The beeswarm plot illustrates that poor adherence assessment consistently drives predictions toward viral nonsuppression (positive SHAP values), while good adherence strongly predicts viral suppression (negative SHAP values). Feature importance rankings (Figure 2C) confirmed adherence assessment as the dominant predictor contributing 54.8% of model gain, with duration on ART (10.2%), age group (8.4%), and urban residence (4.9%) representing secondary but clinically relevant factors. This hierarchy emphasizes adherence as the critical determinant of treatment outcomes, consistent with established clinical understanding that medication compliance fundamentally governs ART effectiveness.

#### Dependence Plots

**Figure 3:**
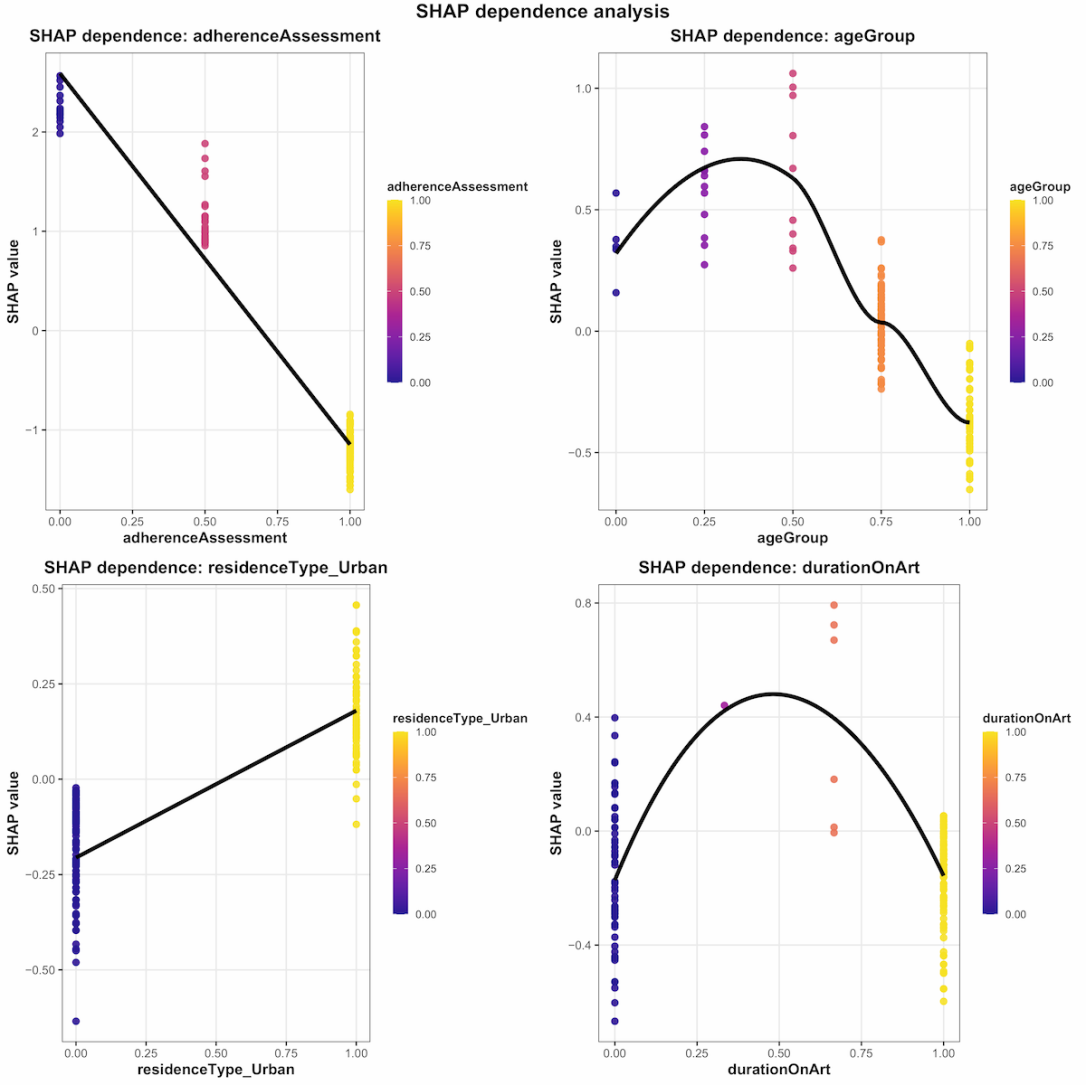
XGBoost dependence plots illustrating the marginal effect of selected predictor variables on the predicted probability of HIV viral suppression. SHAP: Shapley Additive Explanations.

SHAP dependence plots (Figure 3) revealed distinct nonlinear relationships between key predictors and viral nonsuppression risk. Adherence assessment exhibited a clear monotonic relationship, with poor adherence (lower values) consistently increasing SHAP values toward viral nonsuppression predictions, while optimal adherence (higher values) drove predictions toward viral suppression. Age group demonstrated a nonlinear pattern with pediatric and adolescent populations showing substantially elevated risk: young children (aged 0-5 years) exhibited moderately positive SHAP values (~0.5), school-aged children (aged 6-12 years) showed markedly increased risk (SHAP>0.5), while adolescents (aged 13-19 years) displayed the highest predicted nonsuppression risk (SHAP>1.0). Conversely, adults aged 20-35 years demonstrated reduced risk (SHAP<0.5), with those aged 35 years and older showing protective effects (negative SHAP values~–0.1). Residence type displayed a binary pattern where urban residence associated with higher SHAP values (just below 0.5), indicating increased nonsuppression risk compared to rural residence. Duration on ART revealed a complex nonlinear relationship with early treatment periods (3-6 months), showing substantial variability (SHAP values ranging from 0.4 to –0.7), while patients at 7-11 months demonstrated elevated risk (SHAP>0.4). The intermediate period (12-24 months) exhibited the highest predicted nonsuppression risk (SHAP values 0.0 to 0.8), with long-term patients (>24 months) showing predominantly protective effects (SHAP values ranging from 0.1 to –0.6).

### Local Explanation

The analysis encompassed ICEs, breakdown plots, SHAP-based model explainability, force plots, and clustering to provide detailed insights into the model’s decision-making process at the individual level.

#### Individual Conditional Expectations

**Figure 4:**
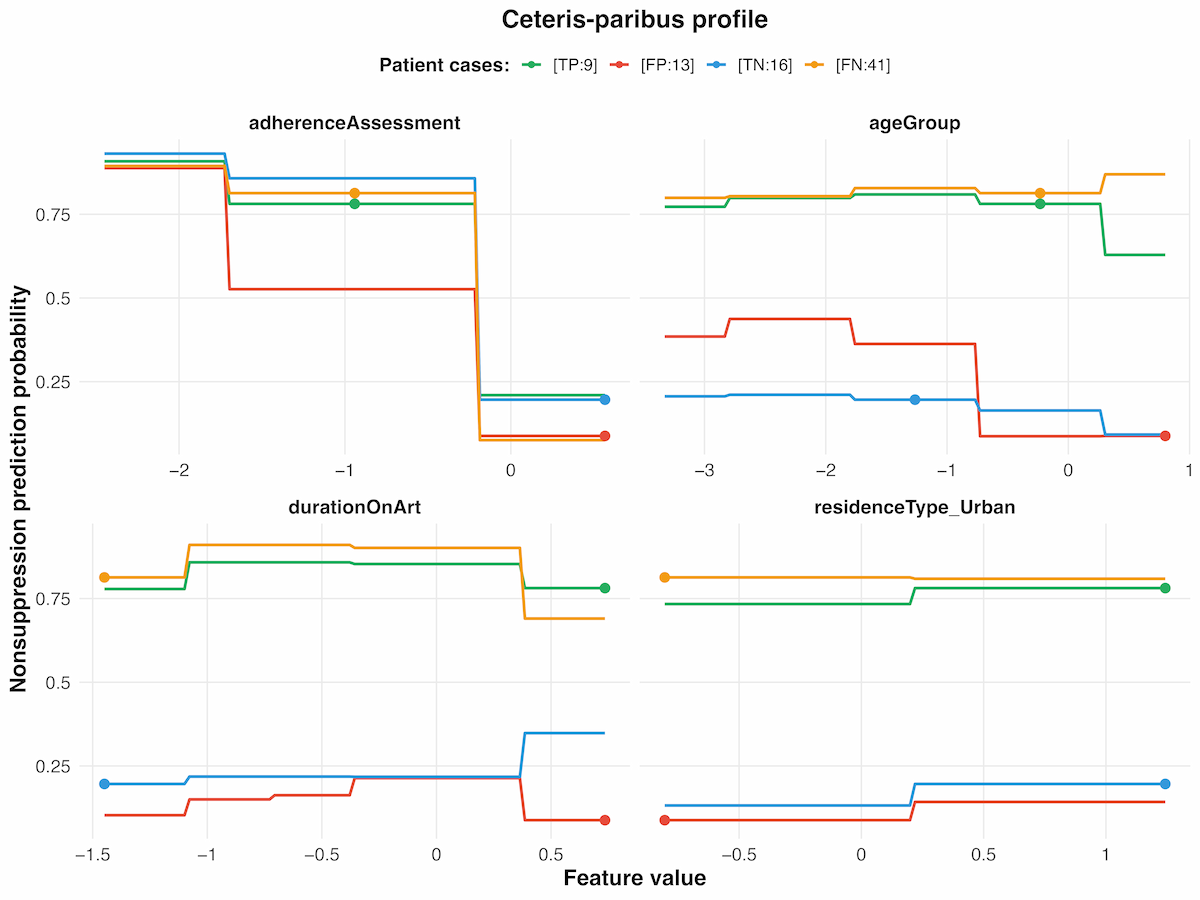
Individual conditional expectation plot using Ceteris-paribus profiles for the XGBoost model. FN:41: false negative, row 41; FP:13: false positive, row 13; TN:16: true negative, row 16; TP:9: true positive, row 9; XGBoost: extreme gradient boosting.

The ICE plot using Ceteris-paribus for the XGBoost model illustrates how 4 key features influenced the model’s nonsuppression predictions for 4 individual cases (Figure 4).

Ceteris-paribus profiles for 4 representative patients (true positive, row 9 [TP:9], false positive, row 13 [FP:13], true negative, row 16 [TN:16], and false negative, row 41 [FN:41]) illustrated distinct individual responses to feature variations across correct and incorrect predictions. The TP case (TP:9) demonstrated a high baseline probability (~0.65-0.70), with adherence assessment showing the steepest probability decline from poor to good adherence, while maintaining elevated risk across most feature combinations. The FP case (FP:13) exhibited moderate baseline probability (~0.25-0.30) with pronounced sensitivity to adherence changes and notable probability elevation at younger age groups, contributing to its misclassification. The true negative (TN) case (TN:16) maintained consistently low probabilities (~0.1-0.2) across all feature variations, with adherence assessment providing the most substantial impact but insufficient to elevate risk substantially. The false negative (FN) case (FN:41) displayed consistently high predicted probabilities (~0.65-0.70) comparable to the TP case across all 4 features, yet was incorrectly classified despite exhibiting similar risk profiles.

#### Breakdown Plots

**Figure 5:**
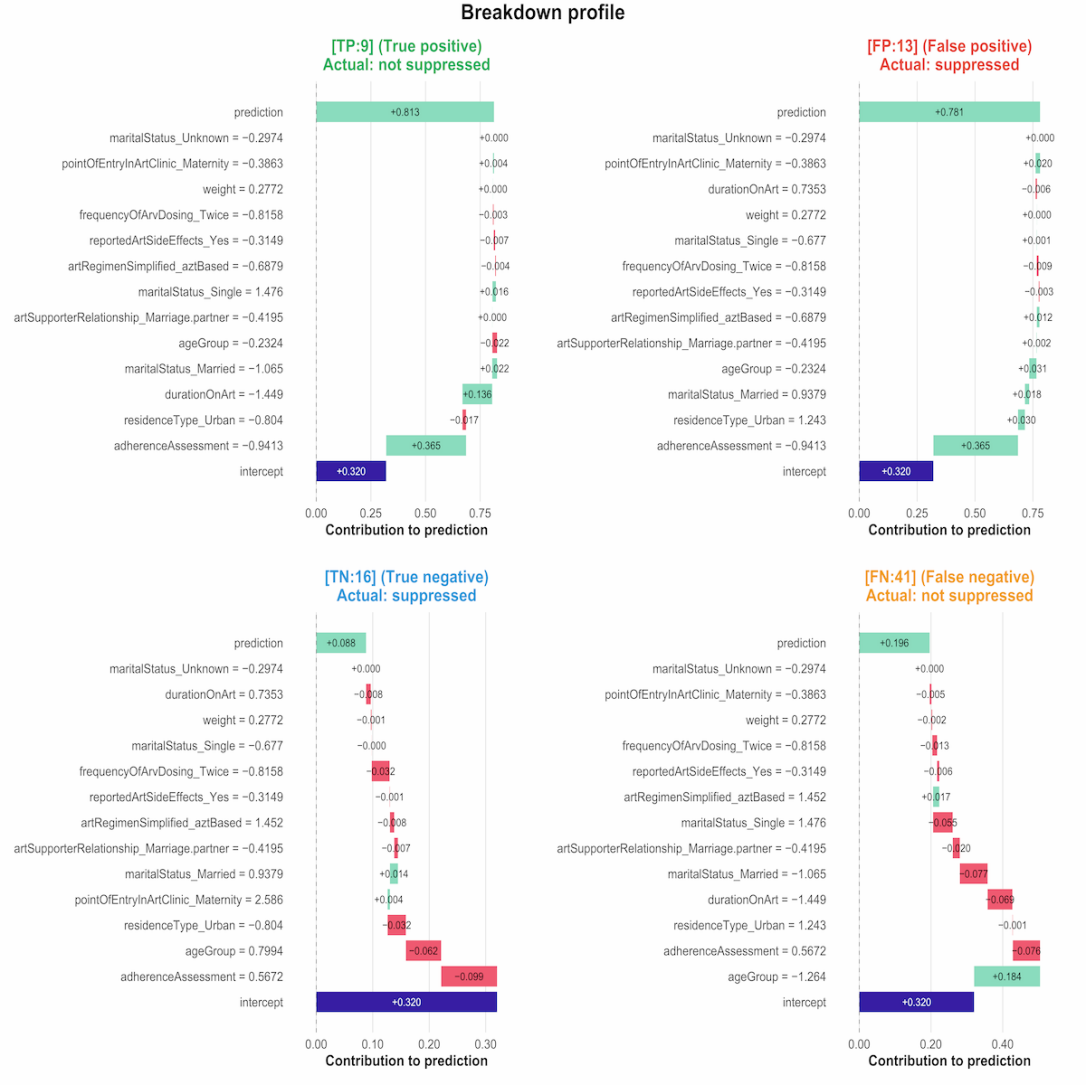
XGBoost breakdown plots for the top 13 features that influenced the prediction outcomes for 4 individual cases. FN:41: false negative, row 41; FP:13: false positive, row 13; TN:16: true negative, row 16; TP:9: true positive, row 9; XGBoost: extreme gradient boosting.

The breakdown plots for individual cases (TP, FP, TN, and FN) illustrate how specific predictors influenced the overall prediction for each observation (Figure 5).

Breakdown plots for the 13 selected features revealed the cumulative contribution of individual predictors to final prediction outcomes across the 4 representative cases. The TP case (TP:9) demonstrated a systematic progression from baseline intercept (0.320) to final prediction (0.813), with adherence assessment providing the largest positive contribution (+0.365), followed by duration on ART (+0.136). Additional features showed mixed effects, with marital status (married) contributing a positive increment, while other features provided negative contributions that partially offset these increases, and the net cumulative effect elevated the prediction above the classification threshold.

The FP case (FP:13) exhibited substantial progression from intercept (0.320) to final prediction (0.781), with adherence assessment dominating the prediction increase (+0.365), followed by age group contributing moderately (+0.031). Marital status (married) and residence type (urban) provided combined positive contributions (+0.048), while other features provided negative contributions that partially offset these increases. Despite the counterbalancing effects of protective features, the model’s final probability assessment substantially exceeded the decision boundary at 0.575, resulting in the misclassification of this actually suppressed patient.

The TN case (TN:16) demonstrated protective feature dominance, with adherence assessment contributing the largest negative effect (–0.099), followed by age group (–0.062) and residence type (urban) (–0.032), while other features provided minimal positive or negative adjustments. The cumulative protective contributions drove the final prediction to 0.088, substantially below the classification threshold, enabling the correct identification of this virally suppressed patient through predominantly risk-reducing feature effects.

The FN case (FN:41) presented a complex feature interaction pattern, with age group providing the primary risk elevation (+0.184), while adherence assessment (–0.076), marital status (married) (–0.077), and duration on ART (–0.069) contributed substantial protective effects. The competing influences of risk-enhancing and protective features resulted in a suppressed final prediction of 0.196, falling considerably below the 0.575 threshold and causing misclassification of this patient with actual viral nonsuppression.

#### Shapley Additive Explanations

**Figure 6:**
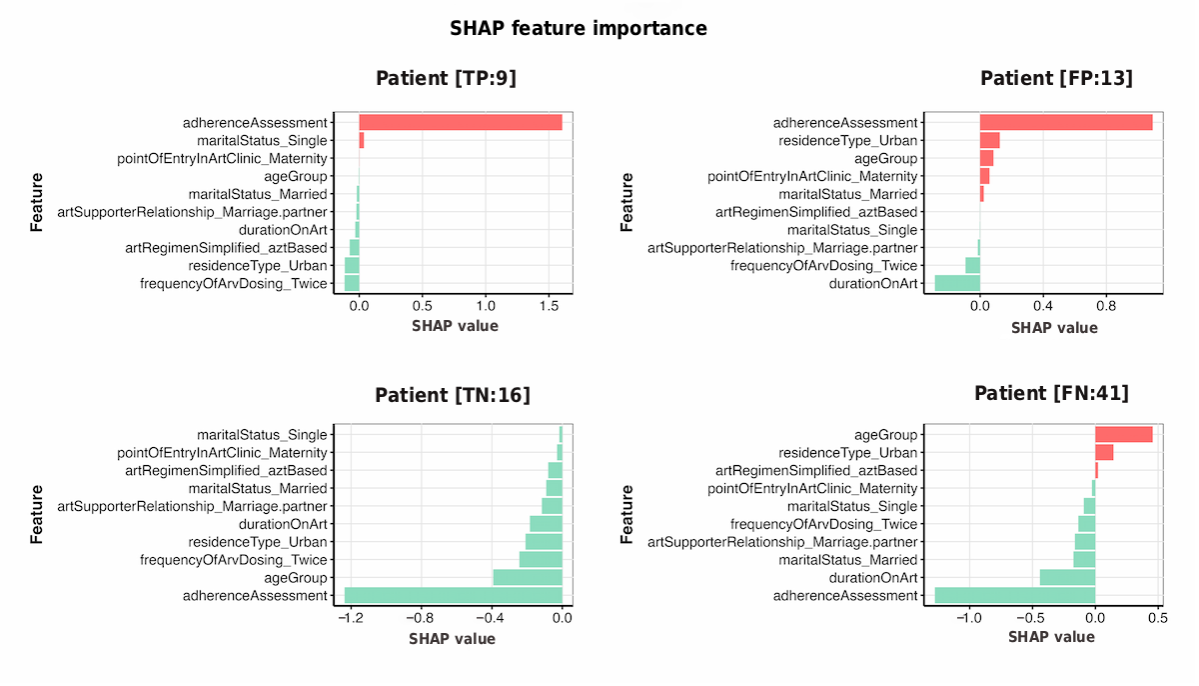
XGBoost SHAP value bar charts for the top 10 features that influenced the prediction outcomes for 4 individual cases. FN:41: false negative, row 41; FP:13: false positive, row 13; SHAP: Shapley Additive Explanations; TN:16: true negative, row 16; TP:9: true positive, row 9; XGBoost: extreme gradient boosting.

The SHAP value bar charts (Figure 6) depict the top contributing features for each individual case (TP, FP, TN, and FN). These plots highlight how key predictors influence the model’s output for viral suppression or nonsuppression predictions.

SHAP value bar charts revealed distinct feature contribution patterns across the 4 representative cases, illustrating individual-level model explanations for each prediction outcome. The TP case (TP:9) demonstrated adherence assessment as the dominant positive contributor (SHAP value>1.5), followed by marital status (single) (~0.1), with most remaining features showing minimal negative contributions close to 0. This pattern indicates that poor adherence primarily drove the model’s prediction toward viral nonsuppression for this correctly identified high-risk patient.

The FP case (FP:13) exhibited adherence assessment as the primary driver (SHAP value~0.9), with residence type (urban) and age group contributing moderately (~0.3), while most other features remained near-neutral with minimal negative contribution from duration on ART. The substantial positive contribution from adherence assessment, combined with other risk factors, elevated the prediction above the classification threshold despite the patient’s actual viral suppression status.

In the TN case (TN:16), adherence assessment emerged as the most significant feature, contributing a negative value of high magnitude (SHAP value: –1.2), followed by age group (SHAP value: –0.4). These contributions reduced the predicted probability of nonsuppression, correctly guiding the model to classify the patient as virally suppressed, consistent with their actual status.

The FN case (FN:41) displayed age group as the strongest positive contributor (SHAP value~0.4), whereas adherence assessment, duration on ART, and marital status showed negative contributions (approximately –1.2, –0.4, and –0.15, respectively). This conflicting pattern of protective features outweighing age-related risk factors resulted in an inappropriately low prediction for a patient with actual viral nonsuppression.

#### SHAP Force Plot

**Figure 7:**
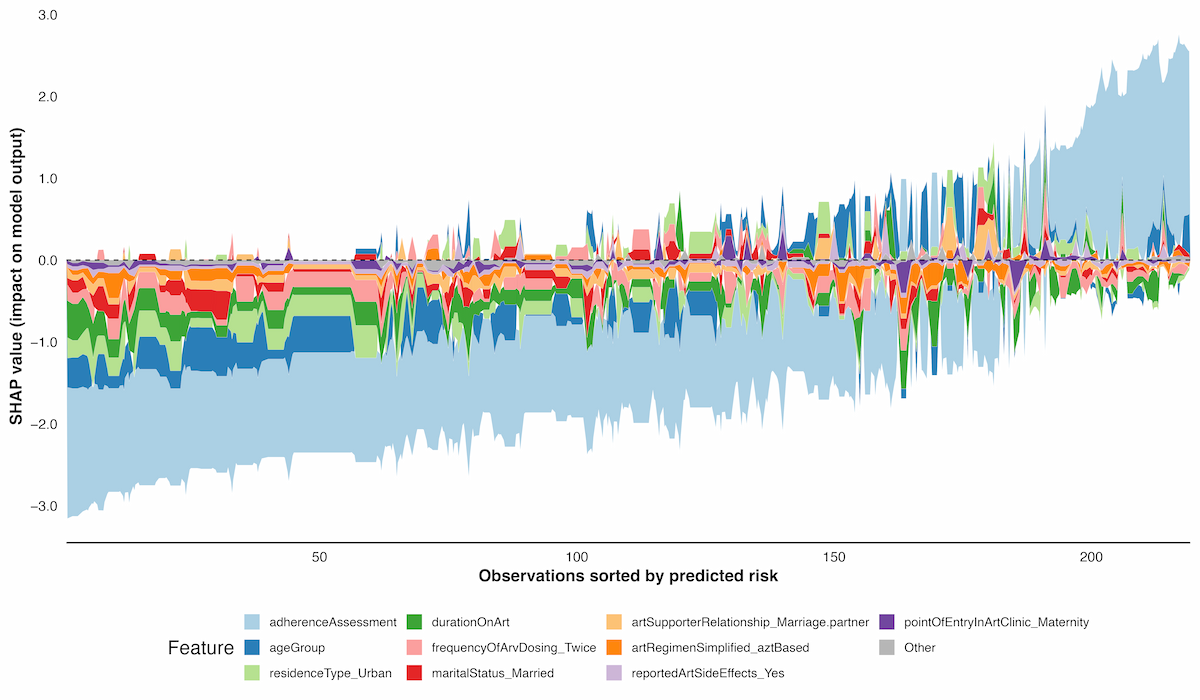
XGBoost SHAP force plot analysis of predictors for HIV viral suppression in Ugandan people living with HIV. SHAP: Shapley Additive Explanations; XGBoost: extreme gradient boosting.

The SHAP force plot (Figure 7) highlights how individual predictors contribute to the model’s predictions of viral suppression or nonsuppression across all observations.

The SHAP force plot revealed feature contribution patterns across all observations, with adherence assessment consistently dominating predictions through substantial positive SHAP values for poor adherence and negative values for good adherence. Age group exhibited nonlinear effects, with pediatric and adolescent ranges generating positive contributions while adult groups provided protective effects. Duration on ART showed variable influences, with intermediate treatment periods contributing to nonsuppression risk and early or long-term durations demonstrating protective effects. Urban residence consistently produced positive SHAP values compared to rural residence, whereas married status typically associated with increased nonsuppression predictions. The visualization effectively demonstrated how competing feature influences determine threshold crossing, revealing the dynamic balance between risk-enhancing and protective factors across individual clinical profiles.

#### SHAP Force Clustering

**Figure 8:**
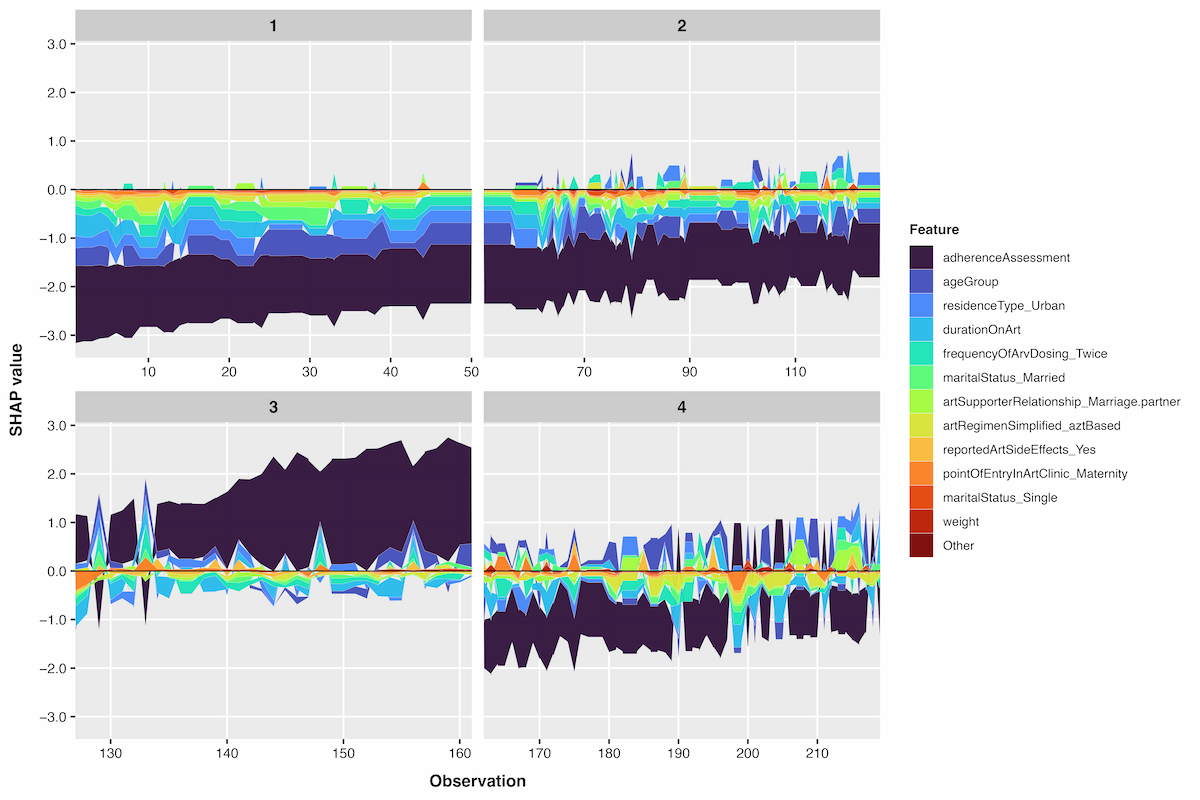
XGBoost SHAP force plot clustering of predictors for HIV viral suppression in Ugandan people living with HIV. SHAP: Shapley Additive Explanations; XGBoost: extreme gradient boosting.

The SHAP force plots (Figure 8) display 4 distinct patient clusters, each characterized by varying influences of key predictors on viral suppression outcomes.

The SHAP force plot clustering revealed 4 distinct patient phenotypes based on feature contribution patterns for viral suppression predictions. Cluster 1 (observations 0-50) demonstrated predominantly protective profiles characterized by substantial negative SHAP values from adherence assessment (>–3) and age group (>–1), with minimal counteracting contributions from other features. This cluster represented patients with good adherence, older age groups, and rural residence, consistently driving predictions toward viral suppression.

Cluster 2 (observations 51-125) exhibited low-risk profiles with negative SHAP values of greater magnitude from adherence assessment (>–2), counterbalanced by moderate positive contributions from other features. This cluster represented patients with good adherence across mixed age groups (young adults and older patients) and diverse residential settings (both rural and urban), with predictions consistently favoring viral suppression despite some offsetting risk factors.

Cluster 3 (observations 126-160) demonstrated predominantly high-risk profiles characterized by substantial positive SHAP values from adherence assessment (>2) and age group, with minimal protective contributions from other features. This cluster represented patients with poor adherence and younger age groups (particularly pediatric and adolescent populations), consistently driving predictions toward viral nonsuppression.

Cluster 4 (observations 161-219) showed variable risk patterns with heterogeneous SHAP value distributions across features, indicating diverse clinical profiles where feature interactions produced inconsistent directional effects. This cluster highlighted the complexity of prediction patterns in patients with mixed risk and protective factors.

## Discussion

This section discusses the implications of our findings on predicting viral suppression in Ugandan people living with HIV on ART. We summarize principal findings, acknowledge study limitations, compare findings with previous research, and discuss the broader significance and potential clinical implications.

### Principal Findings

This study successfully developed an interpretable ML model for predicting viral nonsuppression in Ugandan people living with HIV, achieving robust performance with AUC 0.80, recall 0.65, *F*_1_-score 0.62, and Cohen κ 0.55. The SMOTE-enhanced XGBoost model with XAI techniques revealed critical insights into viral suppression determinants and patient risk stratification.

Adherence emerged as the overwhelming predictor across all analytical approaches, contributing 54.8% of model gain and consistently demonstrating the largest SHAP values. This finding reinforces adherence as the fundamental determinant of treatment success, though the magnitude of its influence suggests that current adherence measurement approaches may inadequately capture the complexity of medication-taking behavior in this population. The model identified a nonlinear age relationship, with adolescents (aged 13-19 years) showing peak nonsuppression risk (SHAP>1.0), declining through young adults, and reaching protective effects in patients aged 35 years and older (SHAP~–0.1). This pattern aligns with known developmental challenges in adolescent HIV care but quantifies the risk magnitude for clinical decision-making.

Urban residence consistently predicted increased nonsuppression risk (SHAP<0.5), despite the dataset’s rural majority. This finding challenges conventional assumptions about health care access advantages and suggests that urban-specific barriers may outweigh accessibility benefits in this population. However, this finding is isolated to this dataset and may not be generalizable to broader contexts.

The intermediate treatment period (12-24 months) emerged as the highest-risk phase, potentially reflecting treatment fatigue or viral resistance development. This temporal vulnerability window has important implications for intensified monitoring and intervention timing.

SHAP clustering revealed 4 distinct patient phenotypes: protective profiles with good adherence and older age (cluster 1), low-risk patients with mixed demographics but good adherence (cluster 2), high-risk adolescents with poor adherence (cluster 3), and complex profiles with variable risk factors (cluster 4). This stratification framework enables targeted intervention strategies aligned with specific risk patterns rather than one-size-fits-all approaches.

### Limitations

The absence of external validation using independent datasets limits confidence in model generalizability beyond the single-site study population at Muyembe HCIV. Routinely collected clinical data introduced several quality threats, including systematic bias from recoding missing values to “N/A” categories and selection bias from analyzing complete records only, which reduced dataset size and potentially excluded patients with complex clinical profiles characterized by incomplete documentation. This approach may have inadvertently favored patients with better health care engagement, limiting model applicability to more vulnerable populations who are typically underrepresented in complete clinical records.

The analysis combined pediatric and adult patients without separate subset evaluation, creating variable coding challenges that potentially compromised model precision. Marriage status proved irrelevant for children, caregiver relationships varied in significance across age groups, and patient weight categories applied uniform standards across vastly different developmental stages. The weight variable’s reduced contribution to model performance likely reflects the complexity of applying standardized categories where weight implications for viral suppression differ substantially between pediatric and adult populations. Additionally, the relatively modest dataset size (N=1101) may have constrained the ensemble algorithm’s ability to capture complex feature interactions, while SMOTE application for class imbalance correction carries overfitting risks if synthetic minority samples inadequately represent true population characteristics.

### Comparison With Prior Work

This study aligns with several investigations that have explored the potential of ML for predicting HIV viral suppression, each possessing its own strengths and limitations [13-18,31]. Various ML algorithms have been used in these studies, with random forest and logistic regression emerging as the most frequently used methodologies.

The findings of Kimaina et al [14] were particularly relevant, as they reported similar performance metrics and the use of ensemble techniques in their analyses. Despite the super learner classifier being identified as the best performer—comprising stacked ensemble models—the XGBoost model demonstrated superior performance compared to other individual algorithms. In our study, we also identified logistic regression and random forest as the top-performing models, following the XGBoost classifier.

A recent study by Seboka et al [16] further emphasized the effectiveness of the XGBoost classifier in predicting viral suppression, identifying critical predictors such as regimen change, adherence level, CD4 lymphocyte count, duration on ART, and tuberculosis status. Though these studies provided insights through global interpretation, they lacked local explanations, limiting the ability to compare individual-level predictions and tailored interventions.

Influential factors in our study reaffirm the critical role of adherence to ART as a pivotal predictor of viral nonsuppression, corroborating previous research that has similarly highlighted its significance in treatment outcomes [17,32-34]. Esber et al [17] demonstrated that adherence, along with CD4 lymphocyte count and ART regimen, was crucial in predicting viral nonsuppression. In contrast, Wagner et al [33] emphasized that viral suppression among participants on dolutegravir is not dependent on strict adherence levels; however, their study also indicated that traditional ART is associated with viral suppression and different adherence levels.

Our identification of age group as a significant predictor aligns with evidence from resource-limited settings. Cross-sectional studies in Cambodia found that older adolescents had a significantly lower likelihood of viral nonsuppression compared to younger peers [35]. This is corroborated by prospective data from Kenya and Uganda, where younger age independently predicted both failure to achieve viral suppression and increased risk of virologic rebound [36]. Population-level data from rural KwaZulu-Natal further support age-related disparities, demonstrating substantially lower viral suppression rates among younger populations, with particular challenges among the younger male population in achieving viral suppression targets [37]. These clinical and population studies emphasize that younger populations require targeted interventions including enhanced psychosocial support and treatment literacy to improve viral suppression outcomes. These findings collectively support age as a critical predictor requiring tailored approaches for younger people living with HIV.

Studies from sub-Saharan Africa show mixed findings, with some demonstrating higher viral suppression in rural areas due to older patient demographics and better ART adherence compared to urban counterparts, while others report better urban outcomes due to improved health care access [38]. Our study found urban residence to be a risk factor for viral nonsuppression, aligning with the former. South African data indicate that virological suppression varied by geographical setting, from 94.6% in urban settings to 88% in rural settings, though this contradicts our findings [39]. In Cameroon, viral suppression was 75% in urban sites compared to 67.7% in rural sites [40]. Our counterintuitive finding may reflect specific urban health care challenges in our setting, including health care fragmentation or urban-specific barriers despite proximity to services. However, this finding is isolated to this dataset and may not be generalizable to broader contexts.

Duration on ART was found to be associated with viral load suppression, with longer durations linked to improved outcomes. This finding is consistent with previous studies [41-43], which highlight that prolonged ART engagement enhances the likelihood of achieving viral suppression. These results reinforce the importance of sustained adherence to treatment in managing HIV effectively.

### Model Interpretability and Clinical Implications

Our primary use of XAI was to identify population-level patterns that inform general clinical and public health strategies. SHAP global feature importance analysis across all 1101 patients revealed that adherence assessment was consistently the strongest predictor of viral nonsuppression, followed by age group, urban residence, and ART duration. These aggregate patterns, derived from the entire cohort, form the basis of our general conclusions about risk factor hierarchies and provide evidence for prioritizing adherence support programs in HIV care settings. Partial dependence plots and ICE curves further confirmed that these relationships held consistently across different patient subgroups, demonstrating robust population-level patterns rather than isolated associations.

While population-level findings drive our general conclusions, individual patient explainability serves 2 critical complementary functions. First, breakdown plots and individual SHAP values validate that population-level patterns manifest consistently at the patient level, ensuring that our aggregate findings are not statistical artifacts but reflect genuine clinical mechanisms. For example, examining individual predictions confirmed that adherence consistently dominated decision pathways across diverse patient profiles, strengthening confidence in our population-level conclusion about adherence primacy. This emphasizes the clinical value of individualized interventions and illustrates how XAI mitigates the inherent black-box nature of ML models by revealing transparent, interpretable decision pathways [44-46].

Second, individual explainability demonstrates clinical applicability by showing how the model functions in practice. Analysis of specific cases, including FPs where poor adherence drove incorrect nonsuppression predictions despite actual viral suppression, illustrates both the model’s reasoning process and its limitations. These examples do not change our population-level conclusions but demonstrate how clinicians might use the model for personalized risk assessment and intervention planning in real-world settings.

This dual approach to explainability fosters accountability and trust in health care artificial intelligence (AI) systems by enhancing comprehensibility at both population and individual levels [47,48]. Transparency in AI decision-making, enabled by XAI, has the potential to build trust among health care professionals and patients alike, facilitating wider adoption of AI-powered health care solutions [45]. Additionally, XAI helps mitigate biases within AI models, promoting fairer and more ethical applications [47-49]. Understanding how various factors influence model predictions allows health care professionals to improve accuracy and ensure that AI-driven decisions align with clinical priorities, thus enhancing the overall utility of AI in health care settings.

This study demonstrates that XGBoost ML models can accurately predict viral nonsuppression in Ugandan patients with HIV, achieving strong discriminative performance (AUC 0.80). XAI analysis identified adherence assessment as the most critical predictor, followed by age group, urban residence, and ART duration. These findings support the integration of ML into clinical decision-making for targeted interventions, particularly adherence support programs for high-risk populations. Future research should focus on external validation across diverse health care settings and the incorporation of additional social determinants of health to enhance model generalizability and clinical utility.

## Data Availability

The data analyzed during this study are available in the Mendeley Data repository [20].

## Appendix

Multimedia Appendix 1 Consolidated reporting guidelines for prognostic and diagnostic machine learning modeling studies.

Multimedia Appendix 2 Map of Bulambuli District, Eastern Uganda, highlighting the primary study site, Muyembe Health Centre IV, and the network of surrounding health facilities providing antiretroviral therapy services.

Multimedia Appendix 3 Supplementary tables detailing variable missingness analysis with exclusion decisions and variable name mapping with descriptions used in the predictive modelling analysis.

Multimedia Appendix 4 Calibration plot for the extreme gradient boosting model predicting viral suppression: incorporating isotonic regression for improved probability calibration.

## Abbreviations

AI: artificial intelligence
ART: antiretroviral therapy
AUC: area under the curve
CD4: cluster of differentiation 4
FN: false negative
FN: 41: false negative, row 41
FP: false positive
FP: 13: false positive, row 13
HCIV: Health Centre IV
ICE: individual conditional expectation
ML: machine learning
SHAP: Shapley Additive Explanations
SMOTE: synthetic minority over-sampling technique
SVM-RFE: support vector machine-recursive feature elimination
TN: true negative
TN: 16: true negative, row 16
TP: true positive
TP: 9: true positive, row 9
WHO: World Health Organization
XAI: explainable artificial intelligence
XGBoost: extreme gradient boosting

## References

[1] (2023). The path that ends AIDS: UNAIDS global AIDS update 2023. UNAIDS.

[2] (2023). Epidemiological fact sheet: HIV statistics, globally and by WHO region. World Health Organization.

[3] Ministry of Health Uganda (2022). Uganda Population-Based HIV Impact Assessment (UPHIA) 2020-2021: summary sheet. PHIA Project.

[4] Lynen L, Van Griensven J, Elliott J (2010). Monitoring for treatment failure in patients on first-line antiretroviral treatment in resource-constrained settings. Curr Opin HIV AIDS.

[5] (2023). The role of HIV viral suppression in improving individual health and reducing transmission: policy brief. World Health Organization.

[6] Ministry of Health Uganda (2022). Consolidated guidelines for the prevention and treatment of HIV and Aids in Uganda - 2022. Uganda National HIV Guidelines.

[7] Panel on Antiretroviral Guidelines for Adults and Adolescents (2024). Guidelines for the use of antiretroviral agents in adults and adolescents with HIV. ClinicalInfo HIV Guidelines.

[8] (2023). EACS Guidelines 2023. Version 12.0. European AIDS Clinical Society.

[9] Joseph Davey D, Abrahams Z, Feinberg M, Prins M, Serrao C, Medeossi B, Darkoh E (2018). Factors associated with recent unsuppressed viral load in HIV-1-infected patients in care on first-line antiretroviral therapy in South Africa. Int J STD AIDS.

[10] Maina E, Mureithi H, Adan A, Muriuki J, Lwembe R, Bukusi E (2020). Incidences and factors associated with viral suppression or rebound among HIV patients on combination antiretroviral therapy from three counties in Kenya. Int J Infect Dis.

[11] Wakooko P, Gavamukulya Y, Wandabwa JN (2020). Viral load suppression and associated factors among HIV patients on antiretroviral treatment in Bulambuli District, Eastern Uganda: a retrospective cohort study. Infect Dis (Auckl).

[12] Rajula HSR, Verlato G, Manchia M, Antonucci N, Fanos V (2020). Comparison of conventional statistical methods with machine learning in medicine: diagnosis, drug development, and treatment. Medicina (Kaunas).

[13] Bisaso KR, Karungi SA, Kiragga A, Mukonzo JK, Castelnuovo B (2018). A comparative study of logistic regression based machine learning techniques for prediction of early virological suppression in antiretroviral initiating HIV patients. BMC Med Inform Decis Mak.

[14] Kimaina A, Dick Jonathan, DeLong Allison, Chrysanthopoulou Stavroula A, Kantor Rami, Hogan Joseph W (2020). Comparison of machine learning methods for predicting viral failure: a case study using electronic health record data. Stat Commun Infect Dis.

[15] Maskew M, Sharpey-Schafer K, De Voux L, Crompton T, Bor J, Rennick M, Chirowodza A, Miot J, Molefi S, Onaga C, Majuba P, Sanne I, Pisa P (2022). Applying machine learning and predictive modeling to retention and viral suppression in South African HIV treatment cohorts. Sci Rep.

[16] Seboka BT, Yehualashet DE, Tesfa GA (2023). Artificial intelligence and machine learning based prediction of viral load and CD4 status of people living with HIV (PLWH) on anti-retroviral treatment in Gedeo zone public hospitals. Int J Gen Med.

[17] Esber A, Dear NF, King D, Francisco LV, Sing'oei V, Owuoth J, Maswai J, Iroezindu M, Bahemana E, Kibuuka H, Shah N, Polyak CS, Ake JA, Crowell TA (2023). Achieving the third 95 in sub-Saharan Africa: application of machine learning approaches to predict viral failure. AIDS.

[18] Mamo DN, Yilma TM, Tewelgne MF, Sebastian Y, Bizuayehu T, Melaku MS, Walle AD (2023). Machine learning to predict virological failure among HIV patients on antiretroviral therapy in the University of Gondar Comprehensive and Specialized Hospital, in Amhara Region, Ethiopia, 2022. BMC Med Inform Decis Mak.

[19] Dwivedi R, Dave D, Naik H, Singhal S, Omer R, Patel P, Qian B, Wen Z, Shah T, Morgan G, Ranjan R (2023). Explainable AI (XAI): core ideas, techniques, and solutions. ACM Comput Surv.

[20] Wakooko P, Gavamukulya Y, Wandabwa JN (2019). Data on viral load suppression and associated factors among HIV Patients on antiretroviral treatment in Bulambuli District, Eastern Uganda. Mendeley Data.

[21] Yang Y (2017). Chapter 4—Ensemble learning. Temporal Data Mining via Unsupervised Ensemble Learning.

[22] Petinrin OO, Saeed F (2019). Stacked ensemble for bioactive molecule prediction. IEEE Access.

[23] Youden WJ (1950). Index for rating diagnostic tests. Cancer.

[24] Zadrozny B, Elkan C (2001). Learning and making decisions when costs and probabilities are both unknown. https://dl.acm.org/doi/10.1145/502512.502540.

[25] Brier GW (1950). Verification of forecasts expressed in terms of probability. Mon Weather Rev.

[26] Lundberg S, Lee SI A unified approach to interpreting model predictions. ArXiv.

[27] Friedman JH (2001). Greedy function approximation: a gradient boosting machine. Ann Stat.

[28] Goldstein A, Kapelner A, Bleich J, Pitkin E (2015). Peeking inside the black box: visualizing statistical learning with plots of individual conditional expectation. J Comput Graph Stat.

[29] Chun MY, Park CJ, Kim J, Jeong JH, Jang H, Kim K, Seo SW (2022). Prediction of conversion to dementia using interpretable machine learning in patients with amnestic mild cognitive impairment. Front Aging Neurosci.

[30] Robnik-Sikonja M, Kononenko I (2008). Explaining classifications for individual instances. IEEE Trans Knowl Data Eng.

[31] Kamal S, Urata J, Cavassini M, Liu H, Kouyos R, Bugnon O, Wang W, Schneider M (2021). Random forest machine learning algorithm predicts virologic outcomes among HIV infected adults in Lausanne, Switzerland using electronically monitored combined antiretroviral treatment adherence. AIDS Care.

[32] Haberer JE, Bwana BM, Orrell C, Asiimwe S, Amanyire G, Musinguzi N, Siedner MJ, Matthews LT, Tsai AC, Katz IT, Bell K, Kembabazi A, Mugisha S, Kibirige V, Cross A, Kelly N, Hedt-Gauthier B, Bangsberg DR (2019). ART adherence and viral suppression are high among most non-pregnant individuals with early-stage, asymptomatic HIV infection: an observational study from Uganda and South Africa. J Int AIDS Soc.

[33] Wagner Z, Wang Z, Stecher C, Karamagi Y, Odiit M, Haberer JE, Linnemayr S (2024). The association between adherence to antiretroviral therapy and viral suppression under dolutegravir-based regimens: an observational cohort study from Uganda. J Int AIDS Soc.

[34] Owusu LB, Ababio C, Boahene S, Zakaria AS, Emikpe AO, Dwumfour CK, Appiagyei KA, Apiribu F (2023). The predictors of unsuppressed viremia among PLHIV: a cross-sectional study in Ghana. BMC Public Health.

[35] Chhim K, Mburu G, Tuot S, Sopha R, Khol V, Chhoun P, Yi S (2018). Factors associated with viral non-suppression among adolescents living with HIV in Cambodia: a cross-sectional study. AIDS Res Ther.

[36] Mujugira A, Celum C, Tappero JW, Ronald A, Mugo N, Baeten JM (2016). Younger age predicts failure to achieve viral suppression and virologic rebound among HIV-1-infected persons in serodiscordant partnerships. AIDS Res Hum Retroviruses.

[37] Otto M, Okango E, Mee P, Dobra A, Tram KH, Gareta D, Otambo W, Moyo R, Sereo T, Blose N, Letoao NS, Mupanguri L, Mwambi H, Herbst K, Tanser F (2025). Trends in population HIV viral suppression: a longitudinal analysis. AIDS.

[38] Shah GH, Etheredge GD, Smallwood SW, Maluantesa L, Waterfield KC, Ikhile O, Ditekemena J, Engetele E, Ayangunna E, Mulenga A, Bossiky B (2022). HIV viral load suppression before and after COVID-19 in Kinshasa and Haut Katanga, Democratic Republic of the Congo. South Afr J HIV Med.

[39] Hermans LE, Carmona S, Nijhuis M, Tempelman HA, Richman DD, Moorhouse M, Grobbee DE, Venter WDF, Wensing AMJ (2020). Virological suppression and clinical management in response to viremia in South African HIV treatment program: a multicenter cohort study. PLoS Med.

[40] Tchouwa GF, Eymard-Duvernay S, Cournil A, Lamare N, Serrano L, Butel C, Bertagnolio S, Mpoudi-Ngole E, Raizes E, Aghokeng AF (2018). Nationwide estimates of viral load suppression and acquired HIV drug resistance in Cameroon. EClinicalMedicine.

[41] Opoku S, Sakyi SA, Ayisi-Boateng NK, Enimil AK, Senu E, Ansah RO, Aning BD, Ojuang DA, Wekesa DN, Ahmed FO, Okeke CB, Sarfo AD (2022). Factors associated with viral suppression and rebound among adult HIV patients on treatment: a retrospective study in Ghana. AIDS Res Ther.

[42] Nega J, Taye S, Million Y, Rodrigo C, Eshetie S (2020). Antiretroviral treatment failure and associated factors among HIV patients on first-line antiretroviral treatment in Sekota, northeast Ethiopia. AIDS Res Ther.

[43] Berihun H, Bazie GW, Beyene A, Zewdie A, Kebede N (2023). Viral suppression and associated factors among children tested for HIV viral load at Amhara Public Health Institute, Dessie Branch, Ethiopia: a cross-sectional study. BMJ Open.

[44] Antoniadi AM, Du Y, Guendouz Y, Wei L, Mazo C, Becker BA, Mooney C (2021). Current challenges and future opportunities for XAI in machine learning-based clinical decision support systems: a systematic review. Appl Sci.

[45] Loh HW, Ooi CP, Seoni S, Barua PD, Molinari F, Acharya UR (2022). Application of explainable artificial intelligence for healthcare: a systematic review of the last decade (2011-2022). Comput Methods Programs Biomed.

[46] Jung J, Lee H, Jung H, Kim H (2023). Essential properties and explanation effectiveness of explainable artificial intelligence in healthcare: a systematic review. Heliyon.

[47] Awotunde JB, Adeniy E, Ajamu GJ, Balogun B, Taofeek-Ibrahim FA (2022). Explainable artificial intelligence in genomic sequence for healthcare systems prediction. Connected e-Health: Integrated IoT and Cloud Computing.

[48] Ali S, Akhlaq F, Imran AS, Kastrati Z, Daudpota SM, Moosa M (2023). The enlightening role of explainable artificial intelligence in medical & healthcare domains: a systematic literature review. Comput Biol Med.

[49] Mathews SM (2019). Explainable artificial intelligence applications in NLP, biomedical, and malware classification: a literature review. Intelligent Computing.

