# Supplementary material for: Explainable AI–Driven Comparative Analysis of Machine Learning Models for Predicting HIV Viral Nonsuppression in Ugandan Patients: Retrospective Cross-Sectional Study
Source: JMIR AI. 2026 Jan 6;5:e68196. doi: 10.2196/68196 (PMC12820540; doi:10.2196/68196)
Supplement: Multimedia Appendix 1 [file ai_v5i1e68196_app1.pdf]

The following is the reporting checklist. A response should indicate whether the particular item is documented in the study. If the response to an item is Y then the location in the article should be provided (e.g., section number), and if the response is N or NA then some reasoning should be provided.

#	Item	Y	N	NA	Location / Reasoning
**Study Details**					
1.1	*The medical/clinical task of interest*	✓			Abstract/Introduction Methods - Predicting HIV viral non-suppression (>1000 copies/mL) in patients on ART
1.2	*The research question*	✓			Abstract/Objectives - Can ML models with XAI accurately predict viral non-suppression and identify key predictors?
1.3	*Current medical/clinical practice*	✓			Introduction - Viral load monitoring per Uganda national guidelines for ART management
1.4	*The known predictors and confounders to what is being predicted / diagnosed*	✓			Introduction/Methods - Adherence, age, urban residence, ART duration, gender, sociodemographic and clinical factors
1.5	*The overall study design*	✓			Methods - Retrospective analysis of secondary data from 2016-2018
1.6	*The medical institutional setting(s)*	✓			Methods - Muyembe Health Centre IV, Uganda
1.7	*The target patient population*	✓			Methods – paediatric and adult population on ART with viral load results at Muyembe HCIV
1.8	*The intended use of the ML model*	✓			Introduction/Methods/Discussion - Clinical decision support for personalized interventions in HIV care
1.9	*Existing model performance benchmarks for this task*	✓			Introduction - Prior ML studies in Eastern/Southern Africa (AUC 0.60-0.9989)

1.10	*Ethical and other regulatory approvals obtained*	✓			Methods – ethical approval was granted by the School of Consumer Intelligence and Information Systems Research Ethics Committee of the University of Johannesburg (Approval Number: 2024SCiiS029).
**The Data**					
2.1	*Inclusion / exclusion criteria for the patient cohort*	✓			Methods - Participants were eligible for inclusion if they were on antiretroviral therapy (ART) for six months or longer and had viral load test results available. All ages were included, covering a wide range of PLWH from paediatric to adult populations.
2.2	*Methods of data collection*	✓			Methods - Clinical and demographic data from 1101 PLWH at Muyembe HCIV (2016-2018)
2.3	*Bias introduced due to the method of data collection used*	✓			Limitations - Selection bias from excluding incomplete records, single rural facility bias
2.4	*Data characteristics*	✓			Results - N=1,101, clinical/demographic characteristics (age, adherence, ART duration, etc.)
2.5	*Methods of data transformations and preprocessing applied*	✓			Methods - Imputation for missing values, ordinal encoding, dummy variables for categorical data
2.6	*Known quality issues with the data*	✓			Limitations - Missing data, inconsistencies in routinely collected health records
2.7	*Sample size calculation*			✓	Not reported in the study, all dataset was used for ML
2.8	*Data Availability*	✓			Methods - Secondary data from Muyembe HCIV clinical records

**Methodology**					
3.1	*Strategies for handling missing data*	✓			Methods - Mean/mode imputation, "Unknown" category for missing categorical data
3.2	*Strategies for addressing class imbalance*	✓			Methods - SMOTE applied to training set for viral non-suppression cases
3.3	*Strategies for reducing dimensionality of data*	✓			Methods- SVM-RFE
3.4	*Strategies for handling outliers*	✓			Normalization
3.5	*Strategies for data augmentation*	✓			Methods - SMOTE for synthetic minority oversampling of non-suppressed cases
3.6	*Strategies for model pre-training*	✓			Not applicable for retrospective study design
3.7	*The rationale for selecting the machine learning algorithm*	✓			Methods - Compared eight ML algorithms; XGBoost selected for superior performance
3.8	*The method of evaluating model performance during training*	✓			Methods - 10-fold cross-validation on training data (80% split)
3.9	*The method used for hyperparameter tuning*	✓			Methods: Grid search for optimizing model hyperparameters
3.10	*Model's output adjustments*	✓			Methods - Threshold optimization for balancing sensitivity and precision
**Evaluation**					
4.1	*Performance metrics used to evaluate the model*	✓			Methods/ Results – Accuracy, precision, recall, specificity, F1-score , AUC , Cohen's kappa
4.2	*The cost or consequence of errors*	✓			Discussion - False positives/negatives impact targeted interventions and resource allocation
4.3	*The results of internal validation*	✓			Results - 80/20 train-test split, XGBoost performance metrics reported
4.4	*The final model hyperparameters*	✓			Results - XGBoost hyperparameters optimized via grid search

4.5	*Model evaluation on an external dataset*		✓		Limitations - No external validation due to single-site study design
4.6	*Characteristics relevant for detecting data shift and drift*		✓		Limitations - Not addressed in the study
**Explainability and Transparency**					
5.1	*The most important features and how they relate to the outcome(s)*	✓			Results - SHAP analysis: adherence (primary), age group, urban residence, ART duration
5.2	*Plausibility of model outputs*	✓			Discussion - Outputs align with clinical understanding of HIV treatment factors
5.3	*Interpretation of model's results by an end-user*	✓			Discussion - XAI (SHAP, ICE plots) enables actionable insights for clinicians
