# Supplementary figures and images for: Explainable AI–Driven Comparative Analysis of Machine Learning Models for Predicting HIV Viral Nonsuppression in Ugandan Patients: Retrospective Cross-Sectional Study
Source: JMIR AI. 2026 Jan 6;5:e68196. doi: 10.2196/68196 (PMC12820540; doi:10.2196/68196)

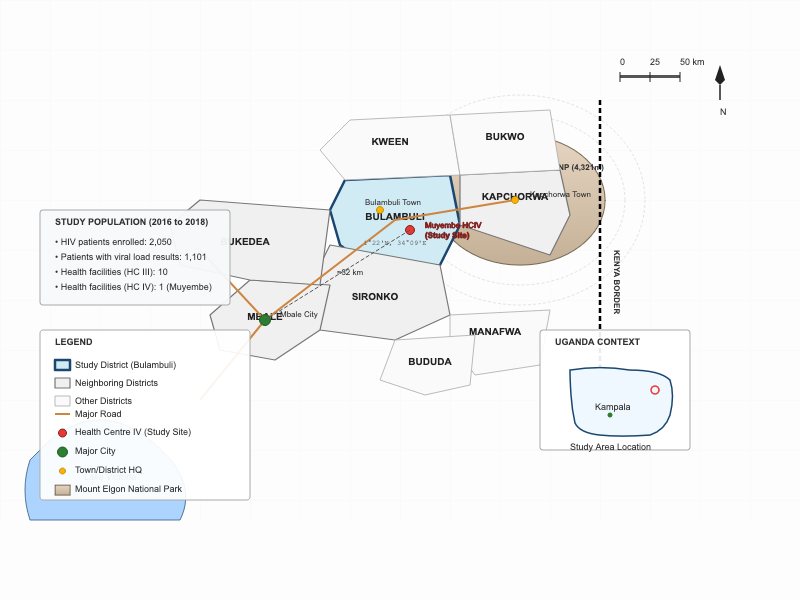

Supplement: Multimedia Appendix 2 [file ai_v5i1e68196_app2.png]

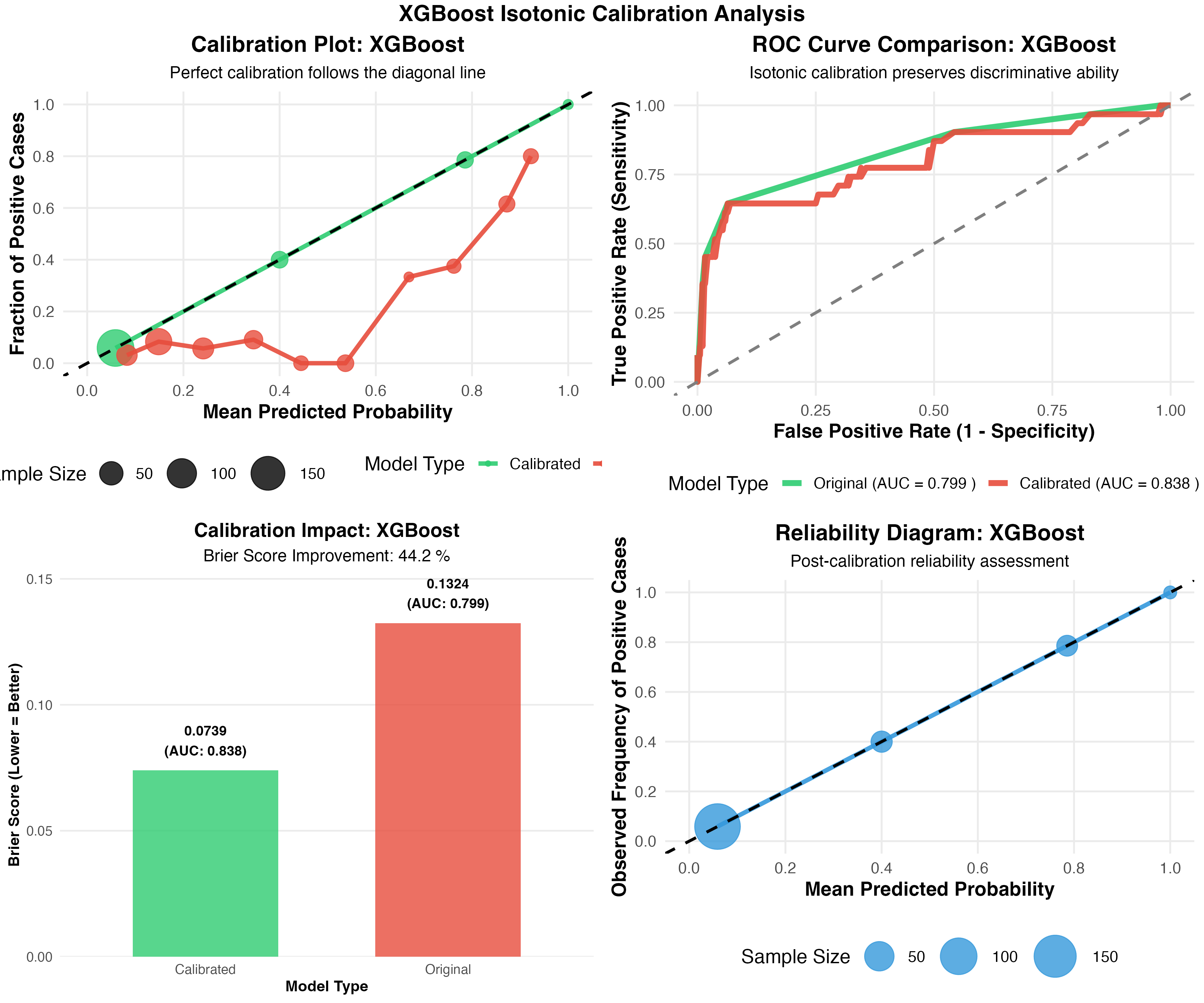

Supplement: Multimedia Appendix 4 [file ai_v5i1e68196_app4.png]
