# Supplementary material for: Explainable AI–Driven Comparative Analysis of Machine Learning Models for Predicting HIV Viral Nonsuppression in Ugandan Patients: Retrospective Cross-Sectional Study
Source: JMIR AI. 2026 Jan 6;5:e68196. doi: 10.2196/68196 (PMC12820540; doi:10.2196/68196)
Supplement: Multimedia Appendix 3 [file ai_v5i1e68196_app3.docx]

**Table S1.** Variable missingness analysis and exclusion decisions.

*Dataset: Muyembe Health Centre IV, Bulambuli District, Uganda (N=1101 patients with viral load results)*

| **Variable** | **Missing (%)** | **Status** |
| --- | --- | --- |
| Age Group | 0.00 | Retained |
| Gender | 0.00 | Retained |
| Marital Status | 9.45 | Retained |
| Residence Type | 0.00 | Retained |
| WHO Clinical Stage | 2.36 | Retained |
| Weight | 0.00 | Retained |
| Opportunistic Infection History | 0.00 | Retained |
| Specific Opportunistic Infection | 93.73 | Excluded |
| Tuberculosis History on ART | 0.00 | Retained |
| Patient State While on ART | 0.00 | Retained |
| Point of Entry in ART Clinic | 0.00 | Retained |
| Duration on ART | 0.00 | Retained |
| Current ART Regimen | 0.00 | Retained |
| Other Medication Use | 0.00 | Retained |
| Specific Other Medication | 97.73 | Excluded |
| Adherence Assessment | 0.00 | Retained |
| ART History | 0.00 | Retained |
| Reason for Stopping ART | 99.64 | Excluded |
| Reported ART Side Effects | 0.00 | Retained |
| Frequency of ARV Dosing | 0.00 | Retained |
| Pre-ART Counselling Status | 0.00 | Retained |
| ART Supporter Presence | 0.00 | Retained |
| ART Supporter Relationship | 6.63 | Retained |
| Viral Suppression Status (Outcome) | 0.00 | Retained |
| Time Before Viral Load Test on ART | 0.00 | Retained |
| CD4 Count at ART Initiation | 0.00 | Retained |
| ART Regimen Simplified | 0.00 | Retained |

**Note:**

Variables with >80% missing data were excluded from analysis due to insufficient information for modelling or reliable imputation.

Excluded variables: Specific Opportunistic Infection (93.73%), Specific Other Medication (97.73%), Reason for Stopping ART (99.64%)

Retained variables with imputation: Marital Status (9.45%), ART Supporter Relationship (6.63%), WHO Clinical Stage (2.36%)

**Table S2.** Variable name mapping and descriptions.

*This table provides a comprehensive mapping between variable descriptions in Table 1, camelCase variable names used in code and visualisations, and original data codes from the source dataset.*

| **Table 1 Description** | **Variable Name (Code/Charts)** | **Original Data Code** | **Description & Levels** |
| --- | --- | --- | --- |
| Age Group | ageGroup | q11 | Patient age categorised into five groups  *Levels: 0-5 years, 6-12 years, 13-19 years, 20-35 years, Above 35 years* |
| Gender | gender | q12 | Biological sex of the patient  *Levels: Male, Female* |
| Marital Status | maritalStatus | q13 | Current marital status of the patient  *Levels: Single, Married, Divorced* |
| Residence Type | residenceType | q14 | Geographic location of patient residence  *Levels: Rural, Urban* |
| WHO Clinical Stage At ART Initiation | whoClinicalStage | q21 | WHO clinical stage at ART initiation  *Levels: Stage 1, Stage 2, Stage 3, Stage 4* |
| Weight At ART Initiation | weight | q22 | Body weight at time of ART initiation  *Levels: 1-20 kg, 21-50 kg, Above 50 kg* |
| Opportunistic Infection History | opportunisticInfectionHistory | q23 | History of opportunistic infections  *Levels: Yes, No* |
| Tuberculosis History On ART | tuberculosisHistoryOnArt | q24 | Tuberculosis during period on ART  *Levels: Yes, No* |
| Point Of Entry In ART Clinic | pointOfEntryInArtClinic | q26 | Clinical service where patient first accessed ART  *Levels: OPD, Maternity, Antenatal Care Service* |
| Duration On ART | durationOnArt | q31 | Length of time patient has been receiving ART  *Levels: 3-6 months, 7-11 months, 12-24 months, More than 24 months* |
| Current ART Regimen Simplified | artRegimenSimplified | q32a | Simplified categorisation of current ART regimen  *Levels: TDF Based, AZT Based, ABC Based, Other ART* |
| Adherence Assessment Last 3 Months | adherenceAssessment | q34 | Patient adherence to ART in previous 3 months  *Levels: Poor <80%, Fair 80-95%, Good >95%* |
| ART History | artHistory | q35 | Whether patient has previous ART experience  *Levels: Yes, No* |
| Reported ART Side Effects | reportedArtSideEffects | q36 | Patient-reported antiretroviral side effects  *Levels: Yes, No* |
| Frequency Of ARV Dosing | frequencyOfArvDosing | q37 | Daily frequency of antiretroviral medication doses  *Levels: Once, Twice* |
| Pre-ART Counselling Status | preArtCounsellingStatus | q38 | Whether counselling was done before ART initiation  *Levels: Yes, No* |
| Treatment Supporter Presence | artSupporterPresence | q39 | Presence of designated treatment supporter  *Levels: Yes, No* |
| Treatment Supporter Relationship | artSupporterRelationship | q39a | Relationship of treatment supporter to patient  *Levels: Care giver, Relative, Peer, Biological parent, Marriage partner* |
| Viral Suppression Status (Outcome) | viralSuppressionStatus | q41 | Viral load suppression status (primary outcome)  *Levels: Suppressed (<1,000 copies/mL), Not Suppressed (≥1,000 copies/mL)* |
| Time Before Viral Load Test On ART | timeBeforeViralLoadTestOnArt | q42 | Duration on ART when viral load test was performed  *Levels: 6 months, 12 months, >12 months* |
| CD4 Count Category | cd4CountAtArtInitiation | q43 | CD4 cell count at ART initiation (cells/μL)  *Levels: Continuous variable categorised as: <200, 200-500, >500* |

**Note:**

The camelCase variable names are used consistently across all figures, supplementary materials, and code documentation. Original data codes (q11-q43) refer to the Stata dataset structure from the source repository.

*TDF: Tenofovir Disoproxil Fumarate; AZT: Zidovudine; ABC: Abacavir*
